# Cosmetic Habits Associated With Breast Cancer in Benin: A Multicenter Case–Control Study

**DOI:** 10.1111/jocd.70639

**Published:** 2026-01-02

**Authors:** Dégboé Bérénice, Moutaïrou Gloria Moutaïrou, Gnangnon Fréddy, Ayinadou Marielle, Azon Kouanou Angèle, Zomalehto Zavier, Tonato Bagnan Angeline, Adégbidi Hugues, Atadokpèdé Félix

**Affiliations:** ^1^ Clinique Universitaire de Dermatologie‐Vénérologie Centre National Hospitalier et Universitaire Hubert Koutoukou Maga Cotonou Benin; ^2^ Faculté des Sciences de la Santé Université d'Abomey‐Calavi Abomey‐Calavi Benin; ^3^ Clinique Universitaire de Chirurgie Viscérale Centre National Hospitalier et Universitaire Hubert Koutoukou Maga Cotonou Benin; ^4^ Clinique Universitaire de Médecine Interne Centre National Hospitalier et Universitaire Hubert Koutoukou Maga Cotonou Benin; ^5^ Clinique Universitaire de Rhumatologie Centre National Hospitalier et Universitaire Hubert Koutoukou Maga Cotonou Benin; ^6^ Service de Gynécologie‐Obstétrique Centre Hospitalier et Universitaire de la mère et de l'enfant Nantes France

**Keywords:** Benin, breast cancer, cosmetics, hair care cosmetics, make‐up products, perfumes, phytoestrogens

## Abstract

**Introduction:**

Breast cancer remains the leading cause of cancer‐related death among women worldwide. An increasing number of studies highlight the contribution of environmental and lifestyle factors, including cosmetic use, in its development.

**Objective:**

To assess the association between cosmetic and dietary habits and breast cancer risk among women in Benin.

**Methods:**

A case–control study was conducted involving 100 women diagnosed with breast cancer and matched 200 controls in the departments of visceral surgery, internal medicine, dermatology‐venereology, and rheumatology at CNHU‐HKM, and the gynecology‐obstetrics department of CHU‐MEL. Data were collected using a standardized questionnaire addressing family history, dietary patterns, and cosmetic product usage.

**Results:**

In multivariate analysis, several cosmetic practices including the use of alkaline soaps (OR_a_ = 7.26; *p* = 0.001), scented body lotions (OR_a_ = 25.90; *p* < 0.001), perfumes (OR_a_ = 30.43; *p* < 0.01), deodorants (OR_a_ = 5.76; *p* = 0.009), shampoos/conditioners (OR_a_ = 31.92; *p* < 0.001), and lipsticks (OR_a_ = 69.12; *p* = 0.018) were significantly associated with increased breast cancer risk. First‐degree family history of breast cancer was associated with more than a threefold increase in risk. Contrary to existing literature, the consumption of soy, beans, and sesame also appeared to be linked to a higher risk of breast cancer in this population.

**Conclusion:**

Our results show a possible association between environmental factors—particularly the use of cosmetic products—and breast cancer. These results underscore a compelling need for a national cosmetovigilance system in Benin and public health initiatives promoting healthier lifestyles, especially among genetically predisposed women.

## 1 Introduction

Breast cancer is one of the most common types of cancer among women worldwide. According to data from the World Health Organization, approximately 2.3 million new cases of breast cancer were diagnosed in 2020, making it the most prevalent cancer among women. Furthermore, around 685 000 women died from the disease in the same year [1]. In Benin, the age‐standardized incidence rate is 22.6 per 100 000 women [2, 3]. Known risk factors for breast cancer include age, family history of the disease, lifestyle, diet, and environmental exposure [4, 5, 6, 7, 8, 9]. In addition to these well‐established risk factors, researchers have also turned their attention to women's cosmetic habits to determine whether these might play a role in the development of breast cancer [6, 10, 11, 12, 13, 14, 15, 16, 17].

Cosmetic products are used daily by millions of women worldwide and contain chemical ingredients such as active agents, preservatives, dyes, fragrances, and solvents, some of which may act as endocrine disruptors or carcinogenic agents [18, 19, 20, 21, 22, 23, 24]. Research on cosmetic habits associated with breast cancer remains relatively limited and results are controversial, with some studies identifying a link between cosmetics and breast cancer, while others find none [5, 6, 12, 13, 14, 15, 16, 17]. Known carcinogenic compounds such as mercury, parabens, and phthalates are banned from cosmetic formulations but are still detected in products sold in some developing countries [16, 22, 23, 25, 26]. Cosmetovigilance systems are almost non‐existent in French‐speaking sub‐Saharan Africa, despite the expansion of the market and the massive importation of cosmetic products. According to our review, studies on cosmetic habits associated with breast cancer are lacking in this region. Our study aims to address this gap by analyzing cosmetic habits among women diagnosed with breast cancer and to identify those associated with an increased risk of breast cancer occurrence in two hospitals in southern Benin in 2023.

## 2 Methods

A prospective, multicenter, observational and analytical case–control study was conducted from July to October 2023 in the departments of visceral surgery, internal medicine, dermatology‐venereology, and rheumatology at CNHU‐HKM and the gynecology‐obstetrics department of CHU‐MEL.

Cases included adult women who were hemodynamically stable, with active breast cancer or a personal history of breast cancer, being followed or previously followed in consultation or hospitalization in the Gynecology‐Obstetrics or Oncology units of the Internal Medicine and Visceral Surgery departments at CNHU‐HKM and CHU‐MEL. The breast cancer follow‐up registry in these hospital centers included a total of 137 patients to be monitored during the study period. Controls were adult women matched to cases within ±5 years of age and ±5 kg of body weight, with no personal history of breast cancer and no current breast cancer, attending consultations in the Rheumatology and Dermatology‐Venereology departments. All cases and controls provided written informed consent. Women who chose to discontinue participation due to discomfort with certain questions or time constraints were excluded. Those with a WHO performance status of 4 or with severe psychiatric disorders were also excluded.

To increase the statistical power, the study followed a 1:2 matching ratio (one case to two controls) and used a non‐probabilistic convenience sampling method. Data collection was carried out using a standardized questionnaire. Data were processed using STATA software for both descriptive and analytical analyses, along with RStudio version 2024.9.0.375. Data organization in tables was performed using Microsoft Excel version 16.90.2. Prior to analysis, data were thoroughly cleaned to correct inconsistencies and missing values. In the descriptive phase, the sample was characterized based on various aspects, including sociodemographic features, family history of breast cancer, alcohol and tobacco use, cosmetic and dietary habits, and lifestyle. For quantitative variables, results were expressed as means with standard deviations for normally or symmetrically distributed data, and as medians with interquartile ranges for non‐normal distributions. Qualitative variables were reported as proportions. In univariate analysis, unconditional binary logistic regression was used, along with bivariate analysis. The significance threshold for all analyses was set at 5% (*p* < 0.05). The associations between identified determinants and the outcome variable were expressed as odds ratios (OR), with confidence intervals. Association measures were presented in tabular format.

The study was conducted in accordance with the principles of the Ethics Committee of the Faculty of Health Sciences of Cotonou and applicable ethical guidelines. Authorization was obtained from the administrations of the participating centers. Each participant received an information sheet, and written informed consent was required prior to questionnaire administration and physical examination. Confidentiality, anonymity, and participants' rights were upheld throughout the study.

## 3 Results

Of the 137 patients initially expected during the study period, 118 actually attended follow‐up outpatient visits or were hospitalized. Eight patients were hospitalized in critical condition, five declined to provide informed consent, and five were unable to complete the interview due to severe psychological distress. In total, we enrolled 100 patients with breast cancer who were followed throughout the study period. The control group consisted of 200 patients. The general characteristics of both case and control populations are presented in Table 1. The most represented age group among both cases and controls was 38–48 years, accounting for 39.00% of the cases and 40.50% of the controls. The mean age of the cases was 44.47 years, with a standard deviation of 11.42 years and a range from 20 to 78 years.

**TABLE 1:** Analysis of the association between sociodemographic characteristics and breast cancer among 300 participants in the 2023 study on cosmetic habits and breast cancer at CNHU‐HKM and CHUMEL.

	Cases		Controls		OR [CI_95%_]	*p*
	*n*	%	*n*	%		
Age (*n* = 300)						
[18–28]	5	5.00	13	6.50	1	
[28–38]	27	27.00	56	28.00	1.25 [0.40–3.90]	0.695
**[38–48]**	**33**	**33.00**	**68**	**34.00**	1.26 [0.41–3.85]	0.682
[48–58]	21	21.00	36	18.00	1.51 [0.46–4.91]	0.484
[58–68]	11	11.00	20	10.00	1.43 [0.39–5.16]	0.583
≥ 68	3	3.00	7	3.50	1.11 [0.19–6.30]	0.902
Occupation (*n* = 300)						
Artisan	9	9.00	33	16.50	1	
Employee	5	5.00	17	8.50	1.07 [0.30–3.76]	0.9
Entrepreneur	1	1.00	29	14.50	0.12 [0.01–1.15]	0.029
Student	1	1.00	14	7.00	0.26 [0.02–2.38]	0.2
Civil servant	18	**18.00**	22	11.00	3.00 [1.10–8.17]	**0.024**
Housewife	23	**23.00**	25	12.50	3.37 [1.27–8.92]	**0.009**
Retired	11	**11.00**	12	6.00	3.36 [1.06–10.65]	**0.028**
Market vendor	32	**32.00**	48	24.00	2.44 [1.01–5.90]	**0.039**

*Note:* Bold values represent the most represented age group among both cases and controls. Proportions of occupations significantly associated with a high risk of breast cancer.

Certain occupations were significantly associated with an increased risk of breast cancer, including government employees (OR = 3, *p* = 0.024), housewives (OR = 3.37, *p* = 0.009), retirees (OR = 3.36, *p* = 0.028), and vendors (OR = 2.44, *p* = 0.039) (Table 1).

Invasive ductal carcinoma was the predominant histological type among cases (68%), followed by unspecified in situ carcinoma and invasive lobular carcinoma (13% each) and finally lobular carcinoma in situ (6%). A positive diagnosis of breast cancer had been established approximately 1 year prior in half of the cases (51%) and between 2 and 3 years earlier in 30% of them.

### 3.1 Associated Cosmetic Habits

Both cases and controls frequently used soaps and body lotions. Among the most commonly used cosmetic products by cases were hair relaxers (78%), antiperspirants/deodorants/perfumes (71%), nail polish (69%), hair pomades (69%), shampoos/conditioners (64%), and makeup products (49%).

In univariate analysis, cosmetics such as deodorants and perfumes, nail polish, makeup products, perfumed body lotions, and alkaline soaps were identified as risk factors, with the risk increased by a factor of 5 to 15 (Table 2).

**TABLE 2:** Analysis of the association between the use of cosmetic products and the risk of breast cancer among 300 participants in the 2023 study on cosmetic habits and breast cancer at CNHU‐HKM and CHUMEL.

	Cases		Controls		OR [CI_95%_]	*p*
	*n*	%	*n*	%		
Soap						
No	0	0	0	0	0	
Yes	100	100.00	200	100.00	—	—
Body lotion						
No	27	27.00	80	40.00	1	
Yes	73	**73.00**	120	60.00	1.80 [1.06–3.06]	**0.027**
Antiperspirants, deodorants, or perfumes						
No	29	29.00	154	77.00	1	
Yes	71	**71.00**	46	23.00	8.19 [4.43–15.14]	**< 0.001**
Makeup products						
No	51	51.00	175	87.50	1	
Yes	49	**49.00**	25	12.50	6.72 [3.59–12.58]	**< 0.001**
Nail polish						
No	31	31.00	174	87.00	1	
Yes	69	**69.00**	26	13.00	14.89 [7.25–30.57]	**< 0.001**
Henna						
No	86	86.00	196	98.00		
Yes	14	**14.00**	4	2.00	7.97 [2.46–25.84]	**< 0.001**
Hair relaxers						
No	22	22.00	176	88.00	1	
Yes	78	**78.00**	24	12.00	26.00 [11.15–60.59]	**< 0.001**
Hair creams or moisturizers						
No	31	31.00	178	89.00	1	
Yes	69	**69.00**	22	11.00	18.00 [8.38–38.65]	**< 0.001**
Shampoos or conditioners						
No	36	36.00	175	87.50	1	
Yes	64	**64.00**	25	12.50	12.44 [6.23–24.85]	**< 0.001**
Hair dyes						
No	82	82.00	192	96.00	1	
Yes	18	**18.00**	8	4.00	5.26 [2.14–12.93]	**< 0.001**

*Note:* Bold values represent proportions of cosmetic products associated with a high risk of breast cancer.

Among the body care products, deodorants, perfumes, nail polish, and makeup products were significantly associated with an increased risk of breast cancer (Table 2). Regarding soaps, both alkaline and skin‐lightening soaps were associated with increased risk (Table 3). All categories of makeup products were implicated (Table 4).

**TABLE 3:** Analysis of the association between the use of different types of soap and the risk of breast cancer among 300 participants in the 2023 study on cosmetic habits and breast cancer at CNHU‐HKM and CHUMEL.

	Cases		Controls		OR [CI_95%_]	*p*
	*n*	%	*n*	%		
Marseille or superfatted soap						
No	79	79.00	133	66.50	1	
Yes	21	21.00	67	**33.50**	0.52 [0.29–0.93]	**0.025**
Caustic soda‐based soap						
No	37	37.00	153	76.88	1	
Yes	63	**63.00**	46	23.12	5.66 [3.20–10.00]	**< 0.001**
Perfumed soap						
No	66	66.00	99	49.50	1	
Yes	34	34.00	101	**50.50**	0.50 [0.30–0.83]	**< 0.001**
Skin‐lightening soap						
No	74	74.00	172	86.00	1	
Yes	26	**26.00**	28	14.00	2.15 [1.17–3.96]	**0.010**
Antiseptic soap						
No	72	72.00	173	86.50	1	
Yes	28	28.00	27	13.50	1.24 [0.71–2.18]	0.440
Traditional soap						
No	75	75.00	134	67.00	1	
Yes	25	25.00	66	**33.00**	2.49 [1.35–4.56]	**0.002**

*Note:* Bold values represent the proportions of soap types shown to be risk factors among cases or protective factors among controls for breast cancer.

**TABLE 4:** Analysis of the association between makeup habits and the risk of breast cancer among 300 participants in the 2023 study on cosmetic habits and breast cancer at CNHU‐HKM and CHUMEL.

	Cases		Controls		OR [CI_95%_]	*p*
	*n*	%	*n*	%		
Foundation						
No	76	76.00	179	89.50	1	
Yes	24	**24.00**	21	10.50	2.69 [1.39–5.18]	**0.002**
Powder						
No	56	56.00	175	87.50	1	
Yes	44	**44.00**	25	12.50	5.50 [2.96–10.18]	**< 0.001**
Lipstick						
No	54	54.00	181	90.50	1	
Yes	46	**46.00**	19	9.50	8.11 [4.12–15.98]	**< 0.001**
Lip gloss						
No	81	81.00	189	94.50	1	
Yes	19	**19.00**	11	5.50	4.03 [1.79–9.02]	**< 0.001**
Eyeliner						
No	81	81.00	189	94.50	1	
Yes	19	**19.00**	11	5.50	4.03 [1.79–9.02]	**< 0.001**
Eye pencil						
Non	76	76.00	183	91.50	1	
Yes	24	**24.00**	17	8.50	3.39 [1.69–6.80]	**< 0.001**
Mascara						
No	83	83.00	187	93.50	1	
Yes	17	**17.00**	13	6.50	2.94 [1.35–6.42]	**< 0.001**

*Note:* Bold values represent the proportions of makeup products reported to be associated with an increased risk of breast cancer.

Among hair products, those significantly associated with breast cancer occurrence included hair relaxers, hair dyes, shampoos and/or conditioners, hair creams, and henna (Table 2). All types of relaxers were implicated, whether lye‐based or not, as well as dark hair dyes, general shampoos, and hair creams (Table 5).

**TABLE 5:** Analysis of the association between hair product use and the risk of breast cancer among the 300 participants in the study on cosmetic habits associated with breast cancer at CNHU‐HKM and CHUMEL in 2023.

	Cases		Controls		OR [CI_95%_]	*p*
	*n*	%	*n*	%		
Hair relaxer use						
None	22	22.00	176	88.00		
With lye	49	**49.00**	16	8.00	24.50 [9.75–61.51]	**< 0.001**
Without lye	29	**29.00**	8	4.00	29 [9.45–88.95]	**< 0.001**
Hair dye use						
None	82	82.00	192	96.00	1	
Light	5	**5.00**	1	0.50	11.7 [1.30–105.37]	**0.005**
Dark	12	**12.00**	1	0.50	28.09 [3.29–239.63]	**< 0.001**
Both	1	100	6	3.00	0.39 [0.04–3.31]	0.371
Shampoo and/or conditioner use						
None	36	36.00	175	87.00	1	
Both (shampoo + conditioner)	46	**46.00**	13	6.50	17.2 [7.33–40.36]	**< 0.001**
Shampoo only	18	**18.00**	12	6.00	7.29 [3.06–17.35]	**< 0.001**
Hair pomades or moisturizing creams						
No	31	31.00	178	89.00	1	
Yes	69	**69.00**	22	11.00	18.00 [8.38–38.65]	**< 0.001**

*Note:* Bold values represent the Proportions of hair care products reported to be associated with an increased risk of breast cancer.

In multivariate analysis, the high‐risk products identified were: alkaline soaps (OR_a_ = 7.26; *p* = 0.001), scented body lotions (OR_a_ = 25.90; *p* < 0.001), perfumes (OR_a_ = 30.43; *p* < 0.01), deodorants (OR_a_ = 5.76; *p* = 0.009), shampoos/conditioners (OR_a_ = 31.92; *p* < 0.001), and lipsticks (OR_a_ = 69.12; *p* = 0.018). These were potential factors associated with an increased risk of breast cancer in this study (Table 6).

**TABLE 6:** Cosmetic habits associated with breast cancer among 300 participants in the study on cosmetic habits linked to breast cancer at CNHU‐HKM and CHUMEL in 2023—Multivariate analysis.

Cosmetic product	Adjusted odds ratio (ORa)	95% confidence interval (CI)	*p*
Soap use			
Alkaline soap	7.26	[2.14–24.57]	0.001
Lotion use			
Perfumed lotion	25.90	[6.52–102.78]	< 0.001
Deodorant and perfume use			
Deodorants	5.76	[1.56–21.26]	0.009
Perfumes	30.43	[6.20–149.32]	< 0.001
Makeup products use			
Lipstick	69.12	[2.09–2277.95]	0.018
Mascara	0.01	[0.001–0.28]	0.006
Shampoos/Conditioners use			
Shampoos and conditioners	31.92	[6.11–166.64]	< 0.001
Shampoos	12.83	[1.62–101.31]	0.015

### 3.2 Other Associated Factors

#### 3.2.1 Family History

Among the cases, 30% had a family history of breast cancer. First‐degree relatives were the most frequently affected, with 30% of patients reporting affected sisters and 20% reporting affected mothers. In the control group, 21.5% had a family history of breast cancer, predominantly among second‐degree relatives: 12% had affected paternal aunts and 10% had affected cousins.

In univariate analysis, having a first‐degree relative (mother or sister) with a history of breast cancer increased the risk of developing the disease by more than threefold (Table 7).

**TABLE 7:** Family history associated with breast cancer among 300 participants in the study on cosmetic habits linked to breast cancer at CNHU‐HKM and CHUMEL in 2023.

	Cases (%)	Controls (%)	Odds ratio [95% CI]	*p*
Any family history of breast cancer				
No	70.00	78.50	1	
Yes	30.00	21.50	1.56 [0.90–2.70]	0.106
First‐degree family history				
Sister	**30.00**	6.98	**3.35**	**0.005**
Mother	**20.00**	16.28		

*Note:* Bold values represent the Proportions of first‐degree family history of breast cancer (mother and sister) among cases.

#### 3.2.2 Dietary Habits

None of the cases smoked, and a quarter of them reported regular alcohol consumption. They regularly consumed phytoestrogen‐rich foods in the following proportions: 90.82% for beans, 73.47% for sesame seeds, and 67.35% for soybeans.

Among the controls, none smoked either, and only 2.5% reported regular alcohol intake. They also regularly consumed phytoestrogens, especially beans (73%) and soybeans (48.5%).

In univariate analysis, the consumption of soybeans, beans, and sesame seeds was associated with increased risk of breast cancer, with the risk being multiplied by factors of 2, 3, and 15, respectively (Table 8).

**TABLE 8:** Consumption of certain phytoestrogens associated with breast cancer among the 300 participants in the study on cosmetic habits linked to breast cancer at CNHU‐HKM and CHUMEL in 2023—Univariate analysis.

	Cases (%)	Controls (%)	Odds ratio [95% CI]	*p*
Soy	67.35	48.50	2.06 [1.25–3.39]	0.004
Beans	90.82	73.00	3.07 [1.53–6.17]	0.001
Sesame	73.47	14.00	15.80 [8.74–28.5]	0.001

In bivariate analysis, the consumption of flax seeds and chickpeas appeared to have a protective effect, whereas the consumption of soybeans, sesame seeds, and beans was associated with an increased risk of breast cancer (Table 9).

**TABLE 9:** Consumption of certain phytoestrogens associated with breast cancer among the 300 participants in the study on cosmetic habits linked to breast cancer at CNHU‐HKM and CHUMEL in 2023—Bivariate analysis.

	Cases		Controls		OR [IC_95%_]	*p*
	*n*	%	*n*	%		
Soy						
No	34	34	103	51.5	1	
Yes	66	**66**	97	48.5	2.06 [1.26–3.42]	**0.004**
Flaxseed						
No	94	94	166	83	1	
Yes	6	6	34	**17**	0.31 [0.11–0.72]	**< 0.001**
Chickpeas						
No	99	99	182	91	1	
Yes	1	1	18	**9**	0.10 [0.01–0.51]	**0.027**
Lentils						
No	100	100	173	86.5	1	
Yes	0	0	26	13	0.00 [0.00–0.00]	**—**
Beans						
No	11	11	54	27	1	
Yes	89	**89**	146	73	2.99 [1.54–6.31]	**0.002**
Sesame						
No	28	28	172	86	1	
Yes	72	**72**	28	14	15. [8.87–29.05]	**< 0.001**

*Note:* Bold values represent the Proportions of phytoestrogen consumption shown to be risk factors among cases or protective factors among controls for breast cancer.

## 4 Discussion

We conducted a case–control study to better identify cosmetic habits associated with breast cancer. The study's statistical power was further enhanced by optimizing the case‐to‐control ratio (1:2) and by selecting controls from the same source population as the cases to minimize selection bias. Data were analyzed using logistic regression. Although methodologically robust, this study is not without limitations. The data collection process relied on participants’ recollection, thereby introducing a potential recall bias. Some odds ratios (notably for hair relaxers and hair dyes) were particularly high, with wide confidence intervals, which warrants cautious interpretation. The marked disparity observed (e.g., 78% exposure among cases vs. 12% among controls for hair relaxers) may reflect a genuine difference in cosmetic habits. However, the fact that the entire questionnaire was self‐reported introduces a potential for error. Specifically, underreporting among controls cannot be entirely excluded, as the definition of exposure (“Yes” vs. “No”) lacked nuance regarding duration and frequency of use, resulting in a strict binary classification. Furthermore, some participants may have been hesitant to disclose certain cosmetic use practices, which could have resulted in social desirability or reporting bias. The findings may also have been influenced by external variables, such as the heterogeneity in the chemical composition of cosmetic products available on the market, as well as the duration and frequency of their use. Although these latter parameters were recorded, they were not reported in the present article. Overall, the data indicated a higher cumulative exposure to cosmetic products among cases, both in terms of years of use and frequency—whether daily, monthly, or annually.

Despite these constraints, appropriate methodological measures were implemented to mitigate these sources of bias and to enhance the validity and reliability of the results.

A population‐based cancer registry study by Joko‐Fru et al. reported a comparable mean age at diagnosis of 44.47 years for breast cancer in sub‐Saharan Africa, reflecting a younger age profile than observed in high‐income countries. Nonetheless, inter‐country variation was noted: the mean age at diagnosis was 46.8 years in Cotonou, 47.3 years in Abidjan, and 45.8 years in Addis Ababa [27].

Occupational categories were found to be significantly associated with breast cancer incidence. This association may reflect differential exposure to carcinogenic agents present in the workplace or domestic environment throughout the life course—either through high‐intensity exposure or cumulative low‐dose exposure. Such agents may include smoke from charcoal or wood, domestic gas, and environmental pollutants, which are known to be prevalent in urban settings across Benin.

Numerous authors have emphasized the critical role of environmental and lifestyle factors in the etiology of breast cancer [6, 7, 8, 9, 28]. In our study, we identified the use of deodorants, perfumes, nail polish, various types of makeup products, scented body lotions, and soaps containing lye. Notably, the use of scented body lotion may be significantly associated with an increased risk of breast cancer (OR = 1.80 [1.06–3.06], *p* = 0.027). Ricketts and subsequently Bastiansz demonstrated that skin‐lightening creams sold in Jamaica contain, on average, higher concentrations of mercury compared to lotions and soaps, thereby exposing users to potentially harmful levels of mercury [21, 22]. Several authors have provided evidence supporting the role of endocrine‐disrupting chemicals (EDCs) and carcinogenic substances found in topical cosmetic products in breast cancer development [10, 11, 13, 15, 16]. The involvement of deodorants and antiperspirants in breast cancer pathogenesis has also been reported in certain studies. This association may be attributed to the presence of aluminum salts, triclosan, and ethylhexylglycerin in these products [12, 29, 30]. It would be crucial to analyze the chemical formulations of deodorants and perfumes available on the Beninese market to identify potential carcinogenic compounds.

Makeup products—particularly foundation, face powder, lipstick, lip gloss, eyeliner, eye pencil, and mascara—were found to potentially be associated with at least a twofold increase in the risk of breast cancer. Findings from Balwierz et al. confirmed the presence of carcinogenic substances—including parabens, ethoxylated compounds, formaldehyde donors, and ethanolamine—in 50 facial cosmetic products sold on the European market [31]. Other researchers have reported contamination of such cosmetic products with heavy metals such as arsenic, cadmium, lead, and mercury [32, 33, 34].

Our findings also revealed that specific hair‐related cosmetic practices showed a possible association with an increased risk of breast cancer. Hair relaxers (whether lye‐based or no‐lye), hair dyes, shampoos and/or conditioners, hair creams, and henna were among the products that could be strongly associated with an increased risk of breast cancer. The use of relaxers, in particular, showed a strong and statistically significant association with breast cancer incidence, regardless of their chemical formulation. Studies conducted in Ghana by Brinton et al. and more recently by Geczik et al. have reported similar findings, highlighting a possible association between the use of hair relaxers—especially lye‐based formulations—and increased breast cancer risk [23, 35]. Regular use of hair relaxers by Black or African American women may pose particular health risks due to potential exposure to estrogen metabolites or endocrine‐disrupting chemicals (EDCs), both of which have been implicated in breast carcinogenesis [14, 17, 36]. Moreover, some researchers suggest that cosmetic products predominantly marketed to and used by Black women may contain higher concentrations of hormonally active compounds [6, 24, 37, 38]. These observations underscore the compelling need for in‐depth investigation into the biological mechanisms linking hair product exposure and breast cancer risk among women of African descent.

The use of hair dyes—especially darker shades—may play a role in the development of breast cancer. Studies conducted in the United States have consistently shown an increased risk linked to the use of hair dyes and chemical straighteners, particularly among Black women [31, 32, 33, 34, 39, 40, 41]. These findings support the hypothesis that chemical constituents in hair products may contribute to breast carcinogenesis.

Hair care products such as relaxers, dyes, lotions, oils, and skin‐lightening creams often contain toxic compounds, including endocrine‐disrupting chemicals (e.g., parabens, phthalates) and known carcinogens. The collective findings from several studies suggest a potential association between regular use of such cosmetic products and an elevated risk of breast cancer [10, 11, 13, 15, 16, 22, 24, 32].

Our study confirmed that genetic factors may play a role in carcinogenesis. Indeed, having a first‐degree relative (mother or sister) with a history of breast cancer was associated with more than a threefold increase in breast cancer risk, consistent with findings reported in the literature [6, 7, 42, 43, 44]. A family history of breast cancer significantly increases a woman's lifetime risk of developing the disease, particularly when the affected relative is a first‐degree family member. Furthermore, a first‐degree family history is a known risk factor for the development of a second primary breast cancer among women previously diagnosed with in situ breast cancer. This risk is especially elevated in women with multiple affected relatives or those with relatives diagnosed before the age of 50 [45, 46, 47, 48]. Women with a significant family history should therefore undergo regular risk assessments and may benefit from tailored screening strategies and genetic testing to enhance risk management [49].

Alcohol consumption, frequently cited as a breast cancer risk factor in the literature, was not identified as significant in our cohort [50]. Conversely, our results indicated that consumption of soy, beans, and sesame may be associated with an increased risk of breast cancer, with respective risk multipliers of 2, 3, and 15. These findings contrast with most of the existing literature, which often suggests a protective role of these foods [51, 52, 53, 54, 55, 56, 57, 58, 59].

These contradictory results may be explained by the fact that our food supply is increasingly contaminated by chemical substances, including fertilizers, pesticides, and metals with harmful effects, which interact with one another and with endogenous proteins and lipids, leading to synergistic or antagonistic effects [60]. Moreover, in our regions, regulations governing the use of phytosanitary products are rarely enforced. Neagu, in a comprehensive review, proposed that environmental exposure to mixtures of chemical xenobiotics can act as a double‐edged sword—either promoting or inhibiting carcinogenesis and breast cancer development [60].

## 5 Conclusion

This study indicates that environmental and lifestyle factors may play a role in breast carcinogenesis. It also highlights that, in Benin, women with a first‐degree family history of breast cancer may bear an additional burden through certain cosmetic practices pursued in the name of beauty.

Our findings are consistent with some previous studies while diverging from others, underscoring the complexity and context‐specific nature of these associations.

It would be prudent to systematically analyze the chemical composition of cosmetic products to identify potential carcinogens and endocrine‐disrupting compounds; establishing a *cosmetovigilance* agency in Benin would allow for the regulation and monitoring of cosmetic product labeling and ingredient transparency. In parallel, preventive strategies should be developed to promote healthier dietary habits and safer cosmetic practices, particularly among women at elevated genetic risk.

## Author Contributions

Prof. Dégboé Bérénice and Prof. Gnangnon Fréddy conceived and designed the study. Dr. Moutaïrou Gloria Moutaïrou served as the principal investigator. Dr. Ayinadou Marielle contributed to drafting the manuscript. Prof. Azon Kouanou Angèle, Prof. Zomalehto Zavier, Prof. Tonato Bagnan Angeline, Prof. Adégbidi Hugues, and Prof. Atadokpèdé Félix facilitated the implementation of the survey and participated in its review.

## Ethics Statement

The study was conducted in accordance with the principles of the Ethics Committee of the Faculty of Health Sciences of Cotonou and applicable ethical guidelines. Authorization was obtained from the administrations of the participating centers. Each participant received an information sheet, and written informed consent was required prior to questionnaire administration and physical examination. Confidentiality, anonymity, and participants' rights were upheld throughout the study.

## Conflicts of Interest

The authors declare no conflicts of interest.

## Data Availability

The data that support the findings of this study are available from the corresponding author upon reasonable request.
